# Managers’ and employees’ experiences of how managers’ wellbeing impacts their leadership behaviours in Swedish small businesses

**DOI:** 10.3233/WOR-220159

**Published:** 2023-05-16

**Authors:** Elena Ahmadi, Daniel Lundqvist, Gunnar Bergström, Gloria Macassa

**Affiliations:** a Department of Occupational Health Science and Psychology, Faculty of Health and Occupational Studies, University of Gävle, Gävle, Sweden; b Department of Behavioural Sciences and Learning, Division of Education and Sociology, Linköping University, Linköping, Sweden; c Unit of Intervention and Implementation Research for Worker Health, Institute of Environmental Medicine, Karolinska Institutet, Stockholm, Sweden; d Department of Public Health and Sports Science, Faculty of Occupational and Health Sciences, University of Gävle, Gävle, Sweden; e EPIUnit–Instituto de Saude Publica, Universidade do Porto, Porto, Portugal

**Keywords:** Leaders, health, wellbeing, SME, managerial behaviours

## Abstract

**BACKGROUND:**

There is a growing interest in managers’ wellbeing due to the observed associations between their wellbeing and leadership behaviours, and between leadership behaviours and employees’ wellbeing. However, it is still unclear how managers’ wellbeing influences their practiced leadership across different workplace contexts, which specific behaviours are affected, and how this varies across time.

**OBJECTIVE:**

The purpose of this study was therefore to explore managers’ and employees’ experiences and perceptions regarding the consequences of managers’ wellbeing for their leadership behaviours in small businesses.

**METHODS:**

Semi-structured interviews were conducted with 39 participants (20 managers and 19 employees) working at 12 Swedish small firms, and analysed using content analysis.

**RESULTS:**

The findings show that managers were more constructive when they felt well, and more passively destructive when unwell. Variations in managers’ wellbeing influenced their mood, energy level, and performance, as well as the company’s working climate. However, these destructive leadership variations did not have a substantial impact, because several protective factors were present.

**CONCLUSION:**

This study shows that the wellbeing of managers in small businesses has perceptible consequences for their leadership behaviours. The study also shows that sustained leadership behaviours may coexist with temporary variations of these behaviours on a constructive-destructive continuum depending on the leader’s wellbeing. Overall, the findings contribute to a more nuanced and dynamic understanding of how the interaction between managers’ wellbeing and their behaviours unfolds in the particular context of small companies.

## 1 Introduction

There is a growing interest in investigating managers’ wellbeing, due to the impact it has on employees and organisations. Systematic reviews indicate associations between managers’ wellbeing and their leadership behaviours [1–3], and between leadership and employees’ health and wellbeing [1, 4–6]. Managers’ leadership may influence employees’ health both directly through their practiced leadership behaviours and indirectly through the working environment and work organisation [4, 7–9]. This makes managers’ wellbeing an important determinant of organisational health.

Previous research has found that transformational leadership and supportive leadership are positively related to employees’ wellbeing [1, 4–6, 10–13]. Further, a number of studies point to relationship-oriented and democratic leadership as being important for employee health and wellbeing, particularly when managers are hands-on and available, show consideration and trust to employees, and empower and motivate employees [10].

Research has generally shown that managers report having good health [14–16] even when experiencing a stressful job [17–20]. However, several recent studies have drawn attention to high levels of stress and sickness absence among managers [21, 22]. Since the role and position of managers within an organisation exert great influence on employees’ health, and given that managers’ wellbeing is an essential prerequisite for their practiced leadership, it is important to know the ways in which managers’ wellbeing impacts their leadership and the workplace in general.

**Fig. 1:**
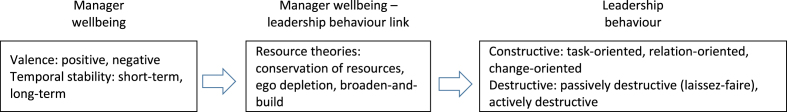
Framework of the link from manager wellbeing to leadership behaviour, after Kaluza et al. [2].

This study investigates the links between managers’ perceived wellbeing and their practiced leadership behaviour, using a conceptual framework developed by Kaluza et al. [2] (Fig. 1). The main elements of the framework (managers’ wellbeing, leadership behaviours and the linkage between them) are explained in detail below. Here, wellbeing is conceptualised broadly as individuals’ judgements about their lives and about feeling well, beyond physical and psychological health [2, 23, 24]. The model differentiates between various facets of wellbeing, such as valence and temporal stability. Valence here refers to positive wellbeing (e.g. work satisfaction, engagement, positive affect) and negative wellbeing (e.g. burnout, job strain, negative affect), while temporal stability is concerned with short-term wellbeing (momentary states, e.g. current affect) and long-term wellbeing (a more stable and lasting state, i.e. life satisfaction) [2, 25].

The framework categorises leadership as constructive or destructive depending on its impact on employees and/or organisational outcomes [2, 26, 27]. Systematic reviews have found a relationship between managers’ positive wellbeing and constructive behaviours, and between their negative wellbeing and destructive behaviours [2, 3].

Constructive leadership has been conceptualised in different ways. One of the approaches appearing in several reviews [2, 12, 26] describes constructive leadership behaviours in the form of three general categories: task-oriented, relation-oriented, and change-oriented leadership behaviours [28–31]. Task-oriented behaviours include planning, clarification of roles and goals, and monitoring operations, and are aimed at effective production and task fulfilment. Relationship-oriented behaviours include providing consideration, encouragement, and recognition, and are focused on relations with employees, support, and empowerment. Finally, change-oriented behaviours include studying the external environment, encouraging and facilitating learning, making innovations in strategies, processes, and products, and viewing problems as opportunities [28–31]. The three dimensions of leadership behaviour are associated with different employee wellbeing and company performance outcomes [4, 5, 13, 32, 33]. In relation to health, however, the strongest association has been found for relation-oriented and change-oriented behaviours [4, 12].

Destructive leadership is a collective name for a wide range of leadership behaviours and styles that are potentially or intentionally harmful for the organisation and/or subordinates [27, 34, 35]. It includes behaviours that are perceived by the employees as systematically hostile, aggressive, and abusive [36]. Leaders being absent, inactive, and avoidant when their active involvement is needed (laissez-faire leadership) [37, 38] is commonly treated as a passive form of destructive leadership [37, 39]. Research has shown that both active and passive forms of destructive leadership are detrimental to both organisational effectiveness and employees’ wellbeing [26, 27, 34, 39–43].

To explain the potential pathways linking managers’ wellbeing to leadership behaviours this study draws on resource theories [2, 44–46]. In conservation of resources theory, wellbeing can be seen as a personal resource that enables managers to function optimally and engage actively in leadership behaviours [44]. Broaden-and-build theory posits that when feeling well, managers may have stronger intellectual, physical, and social resources, leading to broader scope of attention, cognition and action [45]. Finally, ego depletion theory suggests that managers have less energy and recourses to engage in constructive behaviours when they are depleted, and have difficulty suppressing impulses toward negative behaviours when they are tired or irritated [46].

There is a dearth of empirical research investigating the association between managers’ wellbeing and leadership behaviours. The available research has often been conducted using quantitative methods to measure generic leadership constructs such as transformational leadership, leader–member exchange, and destructive leadership [e.g. 47–49]. This makes it hard to distinguish the specific leadership behaviours that are influenced by managers’ wellbeing. Nevertheless, some qualitative studies have investigated experiences of how managers’ wellbeing might influence specific leadership behaviours in concrete workplace contexts. For instance, a study of managers’ experiences in a large industrial Swedish company showed that managers felt more supportive, inspiring, communicating, and available when they were feeling well [18]. Another issue is that the existing research has not addressed the temporal dimension of the relationship between managers’ wellbeing and leadership and how it varies across time. This issue has been raised previously with regard to leadership behaviours (e.g. [50]) and leadership behaviours and employee wellbeing [12].

In the light of the scarcity of the currently available research, there is a need for more knowledge, specifically in the form of qualitative studies on how managers’ wellbeing influences their practiced leadership behaviours across different contexts, which specific behaviours are affected and how this varies across time. Thus, this study addresses the issues mentioned above in the context of small companies due to the large proportion of the workforce employed in small businesses, which in any given society contribute to regional and national economic growth, and also due to the centrality of small businesses managers’ role for organisational health and company survival [51–53]. Despite this, to our knowledge no studies have especially addressed the issue of how managers' wellbeing affects leadership behaviours in the context of small companies. Because of the short distance and social proximity between managers and employees in small companies, the impact of a manager’s wellbeing on their practiced leadership and employees may be more explicit.

The purpose of this study was therefore to explore managers’ and employees’ experiences and perceptions regarding the consequences of managers’ wellbeing for their leadership behaviours in small businesses. The research questions were:

a) How does managers’ wellbeing impact their practiced leadership behaviours, as perceived by the managers and their employees?
b) How does this affect the employees and organisations?

## 2 Materials and methods

### 2.1 Study design and sample

A qualitative approach was chosen since this allows investigation of contextualised phenomena embedded in groups with complex social interaction, where uniqueness is essential [54]. The data were drawn from 39 interviews with managers and employees from 12 small companies.

The companies included in this study were selected within an ongoing regional project called “Successful Companies in Gästrikland” (SCiG), which annually awards the most successful companies in four municipalities in Gästrikland, mid-Sweden. The selection of the nominees is based on economic rating of all the companies in the region according to indicators of profitable growth (such as net sales, number of employees, equity ratio, income, pre-tax profit margin, return on assets, and return on equity) for the last five years. The top 120 companies on the ranking list are then nominated for the award. The full description of the SCiG sample selection can be found elsewhere [55].

For this study, the sample included companies that had been on the project’s nomination list at least once during 2008–2019 (implying that they had demonstrated profitable growth), had 50 employees at most (to correspond to the size of small companies), and had been in continuous operation since 2008. In order to increase variation in the material in terms of degree of sustainable growth, the companies that were placed at the top and bottom of the nomination list for each year during 2008–2019 were contacted. Interviews were conducted until data saturation was reached; this resulted in inclusion of 12 companies, of which nine were at the top of the list and three at the bottom. The nine companies from the top of the list were nominated for the award more than seven times during 2008–2019, indicating sustainable profitable growth in a longer time perspective, and demonstrated increase in net sales and/or number of employees during that period. The three companies from the bottom of the list had a shorter profitable growth during the same period. These companies were nominated only once for the award, and did not demonstrate increase in net sales and/or number of employees during this period.

To gain access to the companies, the CEOs were approached through a letter explaining the purpose of the study and principles of anonymity and confidentiality in treating the data. Additionally, the first author contacted the owner-managers through a telephone call to provide more explanations, agree about the participation and the practical issues. The CEOs then informed their employees about the study, and asked who would be willing to participate in the interviews. The companies’ sizes ranged from 4 to 46 employees, their time in business ranged from 12 to 51 years, and they worked in sales (*n* = 5), manufacturing (*n* = 4), technical consulting (*n* = 2), and transport (*n* = 1).

Participants represented three categories: executive directors (CEOs; *n* = 12; 11 men and 1 woman; 10 owner-managers and 2 non-owners), lower-level managers (*n* = 8; 7 men and 1 woman), and employees (*n* = 19; 15 men and 4 women). Socio-demographic characteristics of the participants are presented in Table 1.

**Table 1:** Characteristics of the participants

	N = 39
Position
CEO	12
Lower-level manager	8
Employee	19
Sex
Male	33
Female	6
Education
Secondary education and similar	28
University education	11
	*mean (range)*
Age, years	43.1 (22–66)
Tenure, years	9.5 (0.1–29)
Manager experience, years	14.4 (2.5–29)

### 2.2 Data collection

The data were collected by means of individual interviews during 2020. A semi-structured qualitative interview guide [54] with a set of topics was employed, and completed with open-ended questions. This allowed respondents to express their thoughts, experiences, and perceptions in a more naturalistic conversation, and allowed the researchers to pose follow-up questions. The related theme in the interview guide concerned consequences of managers’ wellbeing for their leadership behaviours and for their employees. Questions for the employees included “Would you notice if your manager does not feel well? If so, how does it manifest?”, and questions for the managers included “Do you think that your health and wellbeing is important for your leadership? If so, in what way does it affect your leadership?”, and “Can you give an example?”

The interviews were conducted face-to-face by the first author (E.A.) at each company’s premises, and lasted between 60 and 100 minutes. Three interviews were carried out via video conference (Zoom) due to the Covid-19 pandemic. The interviews were digitally audio recorded and then transcribed verbatim, 29 by a professional transcriber and 10 by E.A.

### 2.3 Data analysis

Content analysis was used to identify core meanings in the data which described the phenomenon under study [56]. A conventional inductive approach to content analysis [57], where categories emerge from the data through the researcher’s immersion in and interpretation of the data, was chosen. The analysis followed phases of preparation (selecting the unit of analysis and making sense of the data), organising (open coding, grouping, categorisation, and abstraction), and reporting [58, 59]. Whole interview transcripts were regarded as the unit of analysis, as recommended by Graneheim and Lundman [59], focusing on the manifest content.

The transcribed interview material was read through several times to make sense of the data and to achieve immersion. In the next step, the sentences in the text that seemed to reflect the key thoughts were highlighted and assigned headings. These headings then became the codes that were freely generated during the initial stage of the analysis. The transcribed text was systematically coded to cover all the different aspects of the content in the interview material related to the study’s purpose. Next, the codes that shared similarities were grouped into several broader categories to make meaningful clusters. The primary analysis was performed by the first author (E.A.) in discussion with the second (D.L.). In the process of sorting and abstraction, the researchers repeatedly compared the categories for similarities and differences, building a hierarchy of codes, sub-categories, and categories. Subsequently, the categories were reviewed and discussed with all the research team. Data analysis was carried out in version 9 of ATLAS.ti for Windows (ATLAS.ti Scientific Software Development GmbH, www.atlasti.com), a computer-assisted qualitative data analysis software package.

### 2.4 Ethics

The study was approved by the Swedish Ethical Review Authority (approval number 2019-00314). All participants received an explanation of the purposes of the study and provided informed consent before each interview.

## 3 Results

Two main categories and several subcategories emerged from the analysis of the interviews (Table 2). The results section is divided into two parts corresponding to the main categories. The first part describes the findings regarding the direct consequences of managers’ wellbeing for their behaviours and the organisation, as perceived by the managers and their employees (category 1). The analysis showed that variations in managers’ wellbeing were noticeable in the organisation (1.1), and influenced managers’ mood and energy level, leadership behaviours, own performance, and the workplace climate (1.2).

**Table 2:** Structure of the analysis

Categories	Subcategories
1. Consequences of managers’ wellbeing	1.1. Managers’ wellbeing is noticeable in the organisation
	1.2. Consequences of managers’ wellbeing
	1.2.1. Managers’ mood and energy levels
	1.2.2. Leadership behaviours
	1.2.3. Workplace climate
	1.2.4. Managers’ work performance
2. Factors protecting against negative leadership behaviours caused by managers’ lack of wellbeing	2.1. Importance of managers’ wellbeing and the resultant behaviour changes for employees
	2.2. Perceived protective factors:
	2.2.1. Managers’ stable wellbeing
	2.2.3. Managers’ strategies
	2.2.4. Employees’ strategies

The second part concerns factors that protected the employees and organisations from the negative effects of leadership caused by the managers’ lack of wellbeing (category 2). It became apparent during the analysis that the employees did not experience substantial effects from the negative deviations in leadership behaviour induced by managers’ low wellbeing (2.1), which could have been due to factors (2.2) such as managers’ otherwise-stable wellbeing (2.2.1), and managers’ and employees’ strategies (2.2.3, 2.2.4).

### 3.1 Consequences of managers’ wellbeing

#### 3.1.1 Managers’ wellbeing is noticeable in the organisation

Managers generally reported feeling well, and maintained that employees would instantly notice if they did not, since they usually worked closely together and knew each other well.

“... *We*’*re a small company. We know each other. We know the names of each other*’*s children.* ...  *It becomes a little more personal. You get a little closer to each other. If you have a bad day* ... *it can*’*t go unnoticed. It shows*!” *(respondent* #*18, owner-manager)*

Similarly, employees believed they would notice if their manager did not feel good, because they knew each other well, and met and talked regularly. Nevertheless, some employees in the largest small companies mentioned that they were not sure if they would notice when a manager other than their closest manager did not feel well.

#### 3.1.2 Consequences of managers’ wellbeing

*3.1.2.1 Managers’ mood and energy levels*. The managers said that when they felt well, they had a better mood and more energy, felt happier and more positive, and enjoyed their job more. Employees drew a similar picture, stating that when managers felt well they exhibited a good mood, were more energetic and relaxed, and laughed more often.

When they did not feel well, managers described feeling more tired, broken down, and low on energy; that everything seemed to be heavy and difficult; and that it was not fun to go to work. They became easily irritated and angry, were negative, and complained about small things.

“*Most of the time I*’*m happy and have so much energy, but sometimes it*’*s been tough, and then you get more subdued, and everyone probably sees that at once too.*” *(respondent* #*13, owner-manager)*

Similarly, employees remarked that when managers did not feel well, they exhibited a bad mood, became irritated, and were grumpy.

Generally, managers acknowledged wellbeing to be an important factor for their work.

“*Feeling well is a basic prerequisite for me to be able to do my job, because otherwise it*’*s difficult for me.*” *(respondent* #*9, middle manager)*

*3.1.2.2 Leadership behaviours*. The managers said that when they felt well, they spent more time walking around in the organisation and interacting with employees, and were more available and open for contact. They regarded themselves as more easy-going, easier to cooperate with, more solution-oriented, and more visionary.

“*When I feel well* ...  *then I*’*m probably more on the floor talking to the employees. I have a better structure for how we should move forward, and everything works well. Above all, you*’*re spreading a better energy.*” *(respondent* #*7, middle manager)*

Likewise, the employees said that when managers felt well, they talked more to them, joked, walked around the workplace, and engaged in informal conversations more often. They perceived that their managers were more open and easier to get in contact with.

“*Then it*’*s a happy mood and he comes here and talks more. If he*’*s stressed or under pressure then he mostly sits in the office and closes the door and then he leaves. Otherwise he*’*s out in the store and runs around and talks to everyone, and that*’*s usually the case.*” *(respondent* #*2, middle manager)*

Some employees also described how when their managers felt well, they were more likely to see opportunities and solutions, and presented long-term visions for the company. They also showed more appreciation for their employees’ achievements.

“... *When he*’*s in a good mood and feeling well, then he*’*s that type of person who paints these visions and like: Here we want, and here we go, and this is what we can do*! *And also he*’*s very good at actually telling people that they do things well*... *And the days when he*’*s low or frustrated, he*’*s also quite quick to mention the things that don*’*t work well.*” *(respondent* #*28, employee)*

The managers said that when they did not feel well, they were more withdrawn, more often locked themselves in their office to work, sat for long hours in front of the computer, or worked from home. They kept themselves in their own “bubble”, ate alone, and seldom participated in coffee breaks with employees. They felt that they were less communicative and talked less to their employees. When communicating with employees, they felt that they were not as open and friendly as they used to be, and gave sharper and drier responses. They also felt that they were less attentive, listened less carefully, felt less empathy, and did not have the same understanding for others and their problems as when they felt wellthemselves.

“*If you don*’*t feel well, your energy and attention go inwards to yourself, which means that you become inaccessible, you aren*’*t reachable, you don*’*t have the ability to listen to your employees*’ *challenges, you don*’*t come up with ideas to help them further, you aren*’*t a problem solver. So it affects things fundamentally.*” *(respondent* #*10, CEO)*

Employees gave a similar description of the changes in their managers’ behaviours when they felt ill. They described how their managers were less communicative, gave brief responses, and acted as if they did not have time when employees came and asked questions. The employees experienced that their managers were more inward, shut themselves mentally or physically in their offices, were more difficult to get in contact with, and had fewer spontaneous informal discussions.

Some employees mentioned, however, that their managers’ availability was not really affected, since they were always available and answered questions when needed, albeit in a sharper and less pleasant way. One middle manager noted that when the CEO was not feeling well, they were less sensitive towards employees and wanted to decide everything by themself.

“*The sensitivity and responsiveness disappears*... *then it*’*s just straight forward to the point, this is how I go about it; then the* ‘*we*’ *disappears and only the* ‘*I*’ *remains.*” *(respondent* #*9, middle manager)*

According to some employees, when their managers did not feel well they appeared to be more critical, paying more attention to things that were not working well, and were also more likely to give reprimands.

*3.1.2.3 Workplace climate*. Managers believed that when they felt well, they spread positive energy to others, added energy to the workplace, and made the workplace more fun and pleasant. Conversely, when they did not feel well, they spread negative energy, lowered the mood of others, and affected the workplace climate negatively.

“*It goes hand in hand to some extent. If I feel well, I*’*m in a better mood, then I work better and do a better job. I*’*m also perceived by my colleagues as more easy-going when I feel well. If I*’*m irritated and weighed down over other stuff, then there won*’*t be a good atmosphere, and it gets worse most* ...  *I don*’*t think it might affect my job that much. I do what I have to do regardless. But on the other hand, I think it affects the environment. If I*’*m not happy or am finding things tough, the others will notice this. I may not be as nice to them either. You get a bit, maybe not aggressive. But you get a bit, yes, just grumpy and whiny about little things.*” *(respondent* #*19, owner-manager)*

The employees also said that the managers’ mood was contagious and influenced the atmosphere at the working place both positively and negatively, depending on the managers’ better or worse wellbeing.

“*If they*’*re happy, it*’*s clear that it*’*s much easier to work. If they*’*re in a bad mood, it may be radiating towards us. If they*’*re happy, then that radiates towards us, and it*’*s more fun as well.*” *(respondent* #*39, employee)*

*3.1.2.4 Managers’ work performance*. The managers said that when they were not feeling well, they were less productive, achieved less, and could not handle as many clients as usual. They felt less focused and more distracted, and more often made mistakes and set the wrong priorities. Under these circumstances, they often did only what they had to do and did not take care of more demanding tasks, did not look for better solutions, and did not aim to be extra accurate. When they felt unwell, they found it easier to see problems than solutions.

“*If you feel unwell then you can*’*t bear*... *lose focus*... *don*’*t have the same sharpness*... *And then, then it*’*s difficult to perform at work, of course.* ... *If you feel unwell, you need to spend more time*... *and you lose the strength a bit*... *And then it*’*s also contagious*... *. You might not have the strength to tackle things that need to be done, since it feels hard*... *and it*’*s difficult to focus on what you need to do.*” *(respondent* #*14, middle manager)*

Conversely, when managers felt well, they worked in a more structured way, made quicker decisions, and had an attitude that everything was going better and better and that no problem was too big.

“*If I feel well, I see no obstacles in anything. If I feel well* ...  *then it*’*s hard to see problems in things but then there*’*s always a solution*... *. If I feel unwell, it might be easier to see a problem instead of trying to find a solution to the challenge.*” *(respondent* #*12, middle manager)*

While the managers were quite sure that their wellbeing influenced their performance at work, none of their employees mentioned managers’ work performance as being particularly affected.

### 3.2 Factors protecting against negative leadership behaviours caused by managers’ lack of wellbeing

#### 3.2.1 Importance of managers’ wellbeing and the resultant behaviour changes for employees

While both managers and employees thought that the managers’ wellbeing influenced their mood, leadership, performance, and working climate, neither group perceived that the managers’ wellbeing and resultant behaviour changes had any substantial impact on the employees or the organisation. The respondents mentioned several possible explanations for this, which are summarised in section 3.2.2.

Several employees stated that managers’ wellbeing had a larger impact on the workplace when it was present than when it was absent; that is, it mattered more when the managers felt well. When the manager was happy and energetic, and went around and talked to employees, this contributed to a positive working climate at the workplace. If the manager did not feel well on a particular day, this did not substantially worsen the working situation, but the positive contribution to the working climate vanished.

#### 3.2.2 Perceived protective factors

*3.2.2.1 Managers’ stable wellbeing*. The employees maintained that their managers generally felt well. In their experience, the managers were only stressed during shorter periods of time, for instance because of work peaks or interruptions, and they seldom appeared to really not feel well. Likewise, most of the managers stated that they normally felt well and that their periods of poor wellbeing were rare and short.

Some employees added that when the managers felt ill for longer periods of time, then there was a stronger impact on the employees, but emphasised that this was not their current work situation.

“ ...  *Our former CEO, he obviously didn*’*t feel well and had a lot of problems, and that affected the whole company* ...  *. it was mostly that he was so absent and perhaps didn*’*t make the decisions that needed to be made, and that this was then placed on the rest of us*... *those kind of decisions that perhaps a CEO should actually make. So then quite a lot of responsibility was put on the employees.*” *(respondent* #*31, employee)*

Similarly, another CEO pointed out that his wellbeing had affected his leadership and his job to a greater extent during an earlier period when the company was smaller and he was the only manager, and subsequently was more involved in the whole company and decision-making. He also explained that he had a larger workload and felt ill during longer periods of time.

*3.2.2.2 Managers’ strategies*. Some managers stated that despite not feeling well, they assumed a professional role and did what they needed to do anyway. They tried to keep their spirits up in front of employees in order to avoid spreading negativity, and tried to act positively even when not feeling well.

“*I think I’m good at keeping my spirits up. That is my goal* ...  *because it*’*s difficult to go into things and feel unwell when I work with the staff, but then I always try to go in with a positive attitude, because that*’*s the basis for getting people to open up and talk, instead of going in with your claws straight out, that doesn*’*t really work* ...  *sometimes you can have a very bad day, but then I prefer to stay in the office, I don*’*t go out and start discussing things* ...  *When I come here I put on my* ‘*job hat*’ ...  ” *(respondent* #*9, middle manager)*

Some managers mentioned the importance of remaining calm in interactions with employees, especially when there were problems.

“*You have to keep all the balls in the air all the time. And then you have to be able to handle it in a* ...  *you can never get upset and get angry at someone who*’*s made a mistake or something like that. You must be able to have your peace of mind* ...  *not to flare up when something happens*... *Because I think it*’*s so important that you have the trust of your employees.* ...  *. Because if they couldn*’*t trust me or weren*’*t able to feel calm with me as a leader, it would probably not be much fun to work here either.* ...  *It*’*s important that you convey that there*’*s security in working here. That you don*’*t have to worry about your job every day* ...  ” *(respondent* #*4, manager -owner)*

Most managers admitted that they preferred to withdraw when they did not feel well, working either in their office or at home in order to avoid affecting employees and the workplace climate by spreading negativity.

“*You get more subdued, and everything goes down* ...  *productivity, thinking, finding solutions and ideas depending on how you feel* ...  *. (The respondent talks about private problems, illness and divorce)*... *And some evenings and nights it*’*s difficult to shut down, depending on what you*’*re faced with, what to solve, and you have poor sleep. And sometimes it can be continuous for several days without almost any sleep. And in the end, you*’*re so drained that you can*’*t even handle yourself. And then I usually try to avoid coming here, and then it*’*s better that I work from home or somewhere else if I feel too broken* ...  *. You don*’*t deliver good signals, I think, as a leader. If you come in looking all gloomy and you have no energy, you have no glow, no spark, then you*’*re almost taking away the energy. As soon as you open the door, you have this black cloud over you; not that you*’*re angry or unpleasant, but you*’*re subdued, and then I think there*’*s a few times it*’*s happened, but those times I*’*ve really thought when looking at myself in the mirror,* ‘*Are you going to expose your people to this?*”’ *(respondent* #*13, owner-manager)*

One manager mentioned that he needed to withdraw to cope, so he could avoid anything that might further affect his mood and could have a chance to contemplate the situation and the reasons for his feeling unwell. Several managers maintained that they had learned to cope with stress by accepting the work situation as it was, by viewing problems as normal challenges and tasks to be responded to, and by not letting themselves become very stressed.

*3.2.2.3 Employees’ strategies*. Some employees mentioned that they provided support to managers when they seemed unwell. For instance, they asked how things were going, and showed concern and consideration. Some of them talked to the manager and offered an outlet for them to express their feelings about the problem.

“*But then it usually drains off after a while, so I mean it*’*s nothing more than that. You see, you*’*ve known each other for so long that it*’*s like a relationship at home, you know when to talk and not talk, so it*’*s the same thing when you work closely with someone here, you can feel* ...  *So it doesn*’*t affect me at all, the only thing I can do is that I can try to help and ask if it*’*s something in particular, because sometimes it may be that he just needs to spend some time venting with me to feel better himself too, and that*’*s good*... *we talk very openly with each other so there are no strange things, if he wants to talk I*’*m here, so there*’*s nothing strange about it.*” *(respondent* # *9, middle manager)*

Managers also talked about employees showing consideration and support when the managers felt unwell.

“‘*I see you*’*re stressed,*’ *they say. And that*’*s what I mean, that we have that relationship.*” *(respondent* #*7, middle manager)*

“*People show understanding and say:* ‘*How are you today? How is your back today?*’ ...  *You get so much warmth and energy from the employees because they care about you.*” *(respondent* #*16, middle manager)*

Both managers and employees pointed out that mutual and trustful relationships as well as the close contact between managers and employees were important factors enabling employees to provide support when managers did not feel well. The employees mentioned that they were understanding if the manager had a stressful work situation or sometimes did not feel well and thus behaved differently. They explained that being a small organisation meant that working relationships were close and the distance between employees and managers was short. They maintained that their manager was more like a co-worker and a colleague whom they knew well.

Another strategy that employees often mentioned was that they avoided contacting managers when they thought they seemed unwell; since they knew the manager would feel better soon, they could ask their questions later, or ask somebody else. Some pointed out that they did not want to bother or burden the manager even more. One employee mentioned that he did not expect a good conversation in that situation, and preferred to wait until later. Furthermore, several employees said that it did not matter if the manager was not available and could not help in solving problems temporarily, because their normal regular close interactions meant that questions were immediately answered and problems solved, so there was no backlog of questions that needed urgentsolutions.

## 4 Discussion

The results showed that the wellbeing of the managers influenced their mood and energy level, which in turn influenced their practiced leadership behaviours, the climate of the workplace, and their work performance. In addition, the results indicated that certain protective factors could lessen the negative influence of the managers’ negative leadership behaviours (induced by poor wellbeing) on employees and the organisation.

### 4.1 Perceived consequences of managers’ wellbeing on their behaviours

Mood and energy can be seen as aspects of managers’ wellbeing that are directly noticeable at the workplace, and that influence the other three identified consequences: leadership behaviours, work climate, and managers’ performance.

The study found that managers’ leadership behaviours varied on a scale from more destructive to more constructive, depending on the managers’ wellbeing valence. Hence, when feeling well, managers used more active, supportive, dialog-oriented, and inspiring leadership behaviours. When not feeling well, they appeared more passive (unavailable, avoidant, less engaged in interactions) and more directive and controlling. The results also indicated that variations in managers’ wellbeing influenced the degree of their engagement in primarily relationship-oriented behaviours (e.g. socialising with employees, showing consideration) but also, to some extent, change-oriented leadership behaviours (e.g. being visionary and emphasising solutions instead of problems). Previous research has shown that relationship-oriented and change-oriented behaviours are associated with employees’ wellbeing [1, 4, 6, 10, 12]. Thus, managers’ impaired wellbeing has an immediate effect on leadership behaviours that previous research has shown to promote employee wellbeing.

These behaviours are more energy-consuming than task-oriented behaviours [2, 11, 48] and therefore may be affected the most when managers’ resources are depleted. Managers may refrain from more demanding activities to minimise their net loss of resources. This may also explain why managers tended to use more active behaviours (e.g. walking around and interacting with employees) when feeling well, and more passive behaviours (e.g. being mentally or physically unavailable and withdrawing from contacts) when not feeling well. Further, depleted resources as a result of managers’ lowered ability to self-control and suppress impulses of negative emotions and reactions may clarify why managers would act unpleasantly in communication with employees when not feeling well. The present results make it evident that a manager’s wellbeing is an important resource for their leadership practice, allowing them to engage in leadership behaviours that are more constructive and thus contributing to healthy organisations.

Managers’ wellbeing also affected their own performance, focus, decision-making, and willingness to take care of more demanding tasks. This is in line with previous research suggesting associations between wellbeing and productivity [6, 60–62] in general and for managers [18, 63, 64]. One possible explanation for the results regarding managers’ lower performance might be related to the depletion of personal resources and consequently lower ability to perform effectively and engage in demanding tasks. The results showed that managers’ mood and energy were perceived to be contagious, and influenced employees through the display of leaders’ emotions. A number of previous studies have pointed to crossover emotion contagion within groups [65] and between leaders and group members [66–68]. Further research is therefore warranted to explore the potential cross-over effect of managers’ mood and emotions on the employees’ affective wellbeing, since this effect may be more pronounced in small organisations due to social proximity between managers and employees.

Thus, this study highlights that managers’ leadership behaviours vary due to variations in their wellbeing. In general, our findings confirm the results presented by Lundqvist et al. [18] in relation to how managers’ wellbeing influences their leadership behaviours and work performance. However, the results of our study in addition show the consequences of managers’ wellbeing for employee wellbeing and working climate through emotion contagion. Our study also takes a step forward by embracing the perspectives of both managers and employees. The findings reveal considerable resemblance between managers’ and employees’ perceptions of the importance of managers’ wellbeing for their behaviours and organisation. This strengthens the validity of the results in this study and provides a more complete picture of the studied phenomenon.

The findings also indicate that the size of the workplace and the degree of social proximity between leaders and employees are important for how managers’ wellbeing and its influence on leadership behaviours are perceived in the organisation. For instance, the results suggest that managers’ wellbeing and the resultant behaviour changes were more noticeable within the smaller small companies than in the larger small companies, and when employees and managers worked in close and regular contact.

Finally, the results of our study go beyond previous research by suggesting that the context of small companies and the temporal dimension of leadership behaviours may matter for how managers’ wellbeing and the resultant leadership behaviours actually impact employees. This is discussed in further detail in the next section.

### 4.2 Perceived impact of variations in leadership behaviours due to managers’ wellbeing

The employees and managers in this study did not experience managers’ poor wellbeing to have any substantial impact on the employees’ work or on the organisation in general. This seems surprising in view of the suggested associations between managers’ wellbeing and leadership behaviours [2], managers’ wellbeing and employee wellbeing [4], and leadership behaviours and employee health [1, 4–6]. Several explanations for this result can be offered.

Firstly, according to the results, the wellbeing of the managers generally seemed to be stable; their periods of poor wellbeing and stress were rare and short, thus constituting only temporary variations. In general, the employees highlighted the positive contribution of a manager feeling well. This indicates the importance of managers’ sustained positive wellbeing and the need for better understanding of what resources support managers’ wellbeing in small companies.

Secondly, it can be concluded that it is important to take the temporal aspect of leadership into account since leadership can vary. Here, it is important to consider both momentary and sustained leadership behaviours [50]. Our results show that short-term variations in managers’ wellbeing induce short-term negative-positive fluctuations in leadership behaviours, and that these deviations coexist with constructive leadership prevailing over longer periods.

**Fig. 2:**
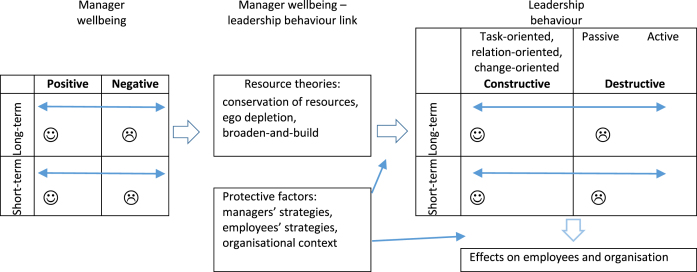
Model of the link from manager wellbeing to leadership behaviour, adjusted from Kaluza et al. [2].

Based on the present results, the initial theoretical framework was extended by adding temporal patterns of leadership and distinguishing between leadership behaviours sustained over longer periods and the day-to-day variations of leadership behaviours, for better representation of the dynamics in the association between managers’ perceived wellbeing and leadership behaviours. In the extended model (Fig. 2), both leadership and wellbeing are represented on a positive-negative scale and in short-term and long-term perspectives. For better visualisation, leadership behaviours are placed on an axis from highly negative to highly positive leadership behaviours; at one end, there are active task-oriented, relation-oriented, and change-oriented leadership behaviours, and on the other end are passive and active destructive leadership behaviours. Task-oriented, relation-oriented, and change-oriented leadership behaviours may also be more or less constructive depending on how actively or passively they are used. Thus, managers’ leadership behaviours may vary daily on the constructive-destructive continuum depending on the momentary state of wellbeing.

The results also indicate that for an individual manager, leadership behaviour that is stable over time may coexist with daily/temporary variations in behaviours on the constructive-destructive continuum, depending on the manager’s own wellbeing. In this study, managers appeared to normally use active constructive behaviours and have stable good wellbeing; however, their daily leadership behaviours could sometimes be passive or even destructive over shorter periods when they did not feel well.

Even if employees did not perceive that the temporary negative variations in leadership had a considerable effect on their work, these variations cannot be disregarded as irrelevant. The short-term variations in leadership are meaningful for a more dynamic description of leaders' behaviours, and could also be relevant for momentary wellbeing of both managers and employees [50]. This may be potentially important if there is a change in the balance of demands (external factors such as the economic situation or the pandemic, or internal private factors such as divorce or the illness of a family member) and resources (e.g. working climate) for managers or employees, which may evoke downstream negative health spirals [69]. Further, it may lead to more sustained negative changes in leadership behaviours due to impaired health resources of managers and/or to negative reactions of employees to the destructive leadership behaviours. Several managers mentioned having experienced longer periods of poor wellbeing a few years back, due to long working hours and work-family conflict. The negative impact on the workplace of them feeling unwell may have been more severe than the impacts described by the study participants.

Thirdly, the results suggest that the consequences of the temporary negative leadership behaviours on employees were counteracted by strategies and protective factors on the level of managers, employees, and organisation. Therefore, these factors were included in our extended theoretical model (Fig. 2). In particular, employees had understanding for managers’ personal and professional situation (which may lessen the importance of the managers’ behaviour changes) and offered consideration and support to the managers (thus increasing the managers’ resources [70]) when managers did not feel well. These strategies built upon organisational factors such as close relationships between managers and employees, good knowledge about each other, regular contacts, and mutual trust, which in turn are often characteristics of small companies [71] or the result of a certain type of work organisation. Employees could also avoid contacts with managers who were not feeling well, which could reduce their exposure to negative leadership behaviours, and instead seek help from others. This was facilitated by factors within the organisational context, such as employees helping each other and regular contacts between managers and employees.

The results also showed that managers used coping strategies (intentional adjustments of their behaviour, e.g. assuming a professional role, trying to be positive, and withdrawing in order to avoid spreading negativity) that could limit the extent to which employees and the company were exposed to the temporary negative leadership behaviours. Viewed through the lens of resource theory [44], coping may presuppose that managers override their impulses to behave negatively and pursue a goal-directed behaviour in spite of lowered resources when they are not feeling well. Exercising self-control is energy-consuming, and requires a large stock of resources [46]. This may indicate that the managers in this study had sufficient resources due to their stable good wellbeing (resource surplus) and probably other protective factors in their organisations to buffer the energy investments in self-regulation.

This implies that managers’ exercising constructive leadership behaviours (overriding impulses to behave negatively despite feeling unwell) provides resources for employees and the company (induced by usage of constructive behaviours), which in turn generates resources supporting managers’ wellbeing (cf. [2]). Hence, the energy invested in suppressing impulses and negative emotions in order to continue exercising constructive leadership behaviours may not lead to future energy loss, due to the positive effects induced by usage of constructive behaviours and their consequences for the employees and the company.

### 4.3 Theoretical and practical implications

This study makes both theoretical and practically applicable contributions to the fields of occupational health, leadership and small business development.

A distinct theoretical contribution of the study is that it responds to the calls for a deeper understanding of the relationship between managers’ wellbeing and leadership behaviours [2] and to managers' health in small businesses [72]. In this regard, the study provides a more nuanced understanding of the implications of the managers’ wellbeing for leadership behaviours and employees wellbeing in the specific context of small businesses.

The results of this study support the otherwise limited previous research on how managers wellbeing affects leadership and which behaviours are affected [2, 18]. The study also deepens this research by drawing attention to (i) the temporal aspect dimension of leadership behaviours and how the perceived association between managers’ wellbeing and leadership behaviours varies across time as well as (ii) how the contextual specificity affects this relationship and the resources within the small companies providing protection from the destructive behaviours. In this regard, these findings develop further the theoretical framework developed by Kaluza et al. [2].

A practical implication of the study is that it draws managers’ attention to the importance of their wellbeing for their leadership behaviour, performance, employees’ wellbeing and, consequently, for the viability and sustainability of businesses. It is therefore essential to raise managers' awareness on this important resource as well as to support them in creating working conditions that diminish strain and promote their own and lower managers’ wellbeing.

The findings also suggest that managers should invest in close and regular contacts with employees and support open climate taking advantage of the manageable company size (small businesses). It is advisable to involve employees and talk openly, not least when the managers do not feel well so that the employees understand their situation better.

### 4.4 Limitations and strengths

There is an obvious limitation concerning the generalisability of the results inherent to any qualitative study. However, companies with different size, age, growth level and from different branches as well as participants with different sex, age, tenure, management levels, and departments, were selected, where possible, to increase variation in context, experiences, and perceptions of the studied phenomenon. This allowed us to elicit richer data, and increased the credibility of the results [56, 59]. Further, the sample size allowed saturation of data when no additional codes and categories appeared, which also increased the trustworthiness of the results [56, 59].

Special sampling within the SGiC project, which implies a focus on companies with profitable growth, might have influenced the result by inducing a positive bias in terms of stronger protective factors that mitigate the negative effect of managers’ changed behaviours; for example, more sustained wellbeing among managers and employees, and a more positive working climate. In order to alleviate this, companies with lower levels of profitable growth were included. However, all the companies in the sample had existed for several years, and hence were rather stable, which could also have influenced the results.

Nevertheless, the study has important strengths. For instance, it uses qualitative methodology which allows to go beyond the predefined categories and explore in more depth the studied phenomenon. In addition, the study combines the perspectives of both managers and employees, which enables to capture more fully the perceived consequences of managers’ wellbeing at the workplace.

## 5 Conclusions

This study shows that the wellbeing of managers in small businesses has perceptible consequences for their leadership behaviours. Variations in managers’ wellbeing influenced their mood and energy level, leadership behaviours, and performance, as well as the company’s working climate. In particular, managers were more constructive when feeling well, and more passively destructive when not feeling well. However, the study also shows that these destructive leadership variations did not have a substantial impact, because several protective factors were present. Sustained leadership behaviours may coexist with temporary variations of these behaviours on a constructive-destructive continuum depending on the leader’s wellbeing.

## Ethical approval

The study was conducted according to the guidelines of the Declaration of Helsinki, and approved by the Swedish Ethical Review Authority (protocol code 2019-00314).

## Informed consent

Informed consent was obtained from all subjects involved in the study.

## Conflict of interest

The authors declare no conflict of interest.
